# Burden, resources, and needs of patients with severe graft-versus-host disease – A qualitative study

**DOI:** 10.1017/S147895152400172X

**Published:** 2025-02-27

**Authors:** Freya Wenzel, Anne Pralong, Christoph Scheid, Marco Herling, Udo Holtick, Steffen T. Simon

**Affiliations:** 1 Department of Palliative Medicine, Faculty of Medicine and University Hospital, University of Cologne, Cologne, Germany; 2 Centre for Integrated Oncology Aachen-Bonn-Cologne-Duesseldorf (CIO ABCD), Faculty of Medicine and University Hospital, University of Cologne, Cologne, Germany; 3 Department of Internal Medicine, Faculty of Medicine and University Hospital, University of Cologne, Cologne, Germany; 4 Department of Hematology, Cellular Therapy, Hemostaseology, and Infectious Diseases, University of Leipzig, Leipzig, Germany; 5 Centre for Health Services Research (ZVFK), Faculty of Medicine and University Hospital, University of Cologne, Cologne, Germany

**Keywords:** Graft-versus-host disease, palliative care, allogeneic hematopoietic stem cell transplantation, quality of life, qualitative content analysis

## Abstract

**Objectives**: Severe forms of acute and chronic graft-versus-host disease (GvHD) are life-threatening complications after adjusted to allogeneic hematopoietic bone marrow or peripheral blood stem cell transplantation (allo-HSCT) and are a major cause of non-relapse mortality. Little is known about the burden, needs, and resources of this specific patient group. This qualitative interview study aimed to explore the experiences of patients with severe forms of GvHD and their perception of palliative care (PC).

**Methods:** Semi-structured interviews were conducted among 13 participants at a tertiary university hospital and were evaluated by qualitative content analysis.

**Results:** The participants described a high psychological and physical symptomatic burden resulting in severely impaired physical function up to loss of independence, which all substantially limited their quality of life (QoL). Frequent long-term hospitalizations highly impacted their social life including the ability to work. A desire to die was frequently experienced, particularly when participants suffered from peaks of burden and uncertainty about the future. Dying was either feared or perceived as relief. Not all participants received PC and the term was sometimes associated with fear or remained unclear to them.

**Significance of results:** Patients with severe forms of GvHD described a multifactorial, high overall burden, and permanently impaired QoL, which needs special support. Next to depressive symptoms, the frequently reported desire to die has not yet been thoroughly studied and requires further research. The infrequent use of PC in this context implicates a need for structural improvement and education in the German healthcare system.

## Introduction

Graft-versus-host disease (GvHD) is a major complication of allogeneic hematopoietic bone marrow or peripheral blood stem cell transplantation (allo-HSCT). It represents a main reason for non-relapse morbidity and mortality (Lee et al. 2003; Zeiser and Blazar 2017). It may manifest as acute GvHD (aGvHD) that is characterized by a rapid and dynamic onset of clinical manifestations and predominantly affects the skin, the gastrointestinal (GI) tract, and liver (Harris et al. 2013). There is also a late-onset form of aGvHD that occurs after day + 100 post-allo-HSCT, and recurrent or persistent forms have been defined (Jagasia et al. 2015). Its prevalence ranges from 40% to 72% in the context of allo-HSCT (Harris et al. 2016).

GvHD can further appear in a chronic form (cGvHD), which is the result of complex allogeneic immune-mediated tissue and organ inflammation, leading to fibrosis and sclerosing processes in various organ systems. About 30–50% of patients suffer from cGvHD after an allo-HSCT (Hamilton 2021).

aGvHD and cGvHD are defined according to severity scores, which are mostly based on organ-specific criteria. Glucksberg et al. categorized aGvHD into 4 severity grades (Glucksberg et al. 1974), which was updated by Harris et al., introducing the MAGIC grading system (Harris et al. 2016). cGvHD is mostly divided into 3 degrees of severity (mild, moderate, and severe), as defined by the NIH Consensus Development Project (Filipovich et al. 2005; Jagasia et al. 2015).

Patients with severe GvHD usually show a high symptomatic burden, which for cGvHD is commonly evaluated by using the Lee Symptom Scale (Teh et al. 2020). Probably due to theirimpaired health condition, the group of patients with severe GvHD is still underrepresented in such analyses (Lee et al. 2018). Thus, in this qualitative study the primary aim was to explore the challenges that those patients face from their own perspective, including their burdens, resources, and needs to give them a voice.

In a previous review, we concluded that special care in the form of multimodal treatment concepts can be helpful for patients with severe forms of GvHD (Wenzel et al. 2021). An important part of this might be the integration of palliative care (PC), since transplant physicians reported GvHD symptoms to be an important indication for the use of PC (El-Jawahri et al. 2018a). Nowadays seen as an integral part of standard cancer care, there is only few data on the role and utilization of PC in the context of allo-HSCT. This indicates that integrating PC into the allo-HSCT procedure likely contributes to achieve the best possible symptom control, to help to better cope with the illness and its prognosis, and to optimize the quality of life (QoL) of affected patients (Simon et al. 2021). Therefore, exploring how the study participants perceive PC was another aim of this study.

## Materials and methods

Using a qualitative approach in the form of a content analysis (Kuckartz 2018), we conducted semi-structured, face-to-face in-depth interviews. Ethical approval was obtained from the Ethics Committee of the Faculty of Medicine of the University of Cologne (Reference Number 20-1253). The reporting of this study follows the guidelines of the Consolidated Criteria for Reporting Qualitative Studies (Tong et al. 2007).

### Recruitment

Patients were recruited face-to-face or via telephone by F.W. (female, medical student) in the form of purposive sampling from October 2020 to March 2021 and were eligible for this study if diagnosed with persistent, severe acute or chronic GvHD after an allo-HSCT. We defined *severe aGvHD* as grade III or IV, following the scoring system by Glucksberg et al. (1974) and s*evere cGvHD* by the NIH Consensus Criteria (Jagasia et al. 2015). Patients were further eligible if they were ≥18 years old, treated in the out- and inpatient bone marrow transplantation (BMT) unit at the University Hospital of Cologne (UHC), with no cognitive impairment (according to clinical assessment) and sufficient German language skills. Verbal and written informed consent for participation in this study was obtained.

#### Data collection

Demographic data and clinical information were retrieved from the patient’s health records of the UHC. The interviews were conducted once by one interviewer (F.W. – trained by S.T.S., male, consultant in PC, senior clinical research fellow) following a semi-structured interview guide (Table 1). The participants did not know the interviewer prior to study commencement. They were informed that the interviewer was working on her doctoral thesis and was not involved in their treatment and that the interviews would be anonymized. The interviews were conducted individually between 2 months and 7 years after transplantation. Whether patients had received PC during the allo-HSCT process varied. Psychological support was offered after every interview if participants felt burdened.

**Table 1.** Topics of the semi-structured interview guide

- Specific symptoms and symptomatic burden of GvHD - Physical and psychological burden - Thoughts about the end of life - Use of palliative care - Patient’s resources - Unmet needs and wishes of patients

### Data analysis

Descriptive analysis was performed for demographic and clinical data of the participants. For analyzing the audio-recorded interviews, the qualitative content analysis by Kuckartz (2018) was used. In this qualitative thematic analysis, categories are formed within a category system (Kuckartz 2019). After verbatim transcription (Institut für Kulturanthropologie und Europäische Ethnologie 2022), the interviews were read several times to get an overview of their contents. The transcripts were not returned to the participants for feedback. By using the software MAXQDA 2020®, the main categories were built deductively, following the semi-structured interview guide. These main categories were coded in all interviews and then divided into subcategories (Table 2), which was performed by 2 researchers (F.W. and Isabel Düster (I.D.); female, research assistant, and medical student – both trained by S.T.S.) and then compared and standardized by developing a description of the coding tree. The following 3 interviews were coded by both researchers to verify the category system. Findings were interchanged regularly among the research team (F.W., Isabel Düster (I.D.), S.T.S.) and modifications were taken to form a final category system. All interviews were then coded. Every code was summarized and integrated into thematic summary tables, which allowed to compare and contrast statements of different participants (Kuckartz 2019).

**Table 2.** Category system – overview of the main categories with first subcategories

**1 Special aspects of the course of the disease**
**2 Burden**
2.1 Due to the general course of the disease
2.2 Specifically due to GvHD
**3 Comprehensive consequences of the illness and its burdens**
3.1 Coping with everyday life
3.2 Social environment
3.3 Being aware of the disease
3.4 Decrease in quality of life
3.5 Other
**4 Perception/experiences during medical care**
4.1 Negative experiences during medical care
4.2 Medical care perceived positively
4.3 Other
**5 Resources/coping factors**
5.1 Structural support
5.2 Mental resources/relief
5.3 Social resources/relief
5.4 Building a feeling of security
5.5 Spiritual resources
**6 Dying/death**
6.1 Confrontation with threat to live/death
6.2 Palliative care
**7 Needs**
7.1 Needs in context of therapy
7.2 Social needs
7.3 Psychological needs
7.4 Physical needs
7.5 Structural needs
**8 Other/individual aspects**

## Results

Seventeen patients were approached, of whom 2 were not interested in participating, 1 felt too ill, and 1 consented, but died before the interview. Thirteen patients (female *n* = 5, male *n* = 8, median age 49 years) were included in the study, until content saturation was assumed (see Table 3 for participant demographics). Two interviews were conducted at the participant’s homes, 2 were conducted during inpatient stays, and 9 were conducted ambulatory. They lasted 38–72 min and during 2 interviews a family member was present.

**Table 3.** Demographic data of participants

Characteristics	Participants (*N* = 13)
Gender	
Female	5
Male	8
Median age (years)	49
18−39	3
40−59	8
>60	2
Primary disease	
Acute myeloid leukemia	9
Acute lymphoblastic leukemia	1
Chronic lymphocytic leukemia	1
Myelodysplastic syndrome	1
Myeloproliferative disorder	1
Characteristics of GvHD	
Acute: overall grade 4	6
Main organ site: lower gastrointestinal tract	6
Chronic: NIH grade severe	7
Main organ site: lung	1
Lower gastrointestinal tract	4
Skin	1
Fasciitis/tendinitis	1
Family status	
Married/long-term relationship	8
Single/widowed	5
Having children	
Non-adult	6
Adult (≥18 years)	3
None	4
Occupation (if have worked before allo-HSCT)	
Full-time	1
Part-time	3
None	8
Time post-allo-HSCT (years)	
0−2	5
2−4	3
4−6	4
6−7	1
Overall duration of cumulative inpatient stay(s) prior to the time of interview (days)	
30−60	2
61−90	4
91−120	2
120−200	5

### Burden associated with specific forms of severe GvHD

#### Severe GvHD of the GI tract

The majority of the participants suffered from a severe aGvHD of the GI tract and named diarrhea and pain as the 2 major burdensome symptoms.

I had only been laying on the couch, because, I had partly … to go to the toilet twice per hour. And this continuously, during day and night-time. (P10, Pos. 15)

The frequency and persistence of diarrhea led to great exhaustion, involving bedriddenness, and unpredictably long hospital stays with dependence on healthcare providers (HCPs) or patient’s social network. Commonly experienced fecal incontinence caused distress and insecurity, leading to avoidance of social contact. Suffering from nocturnal diarrhea was mentioned as an important reason for insomnia. In the course of GvHD, many participants experienced a decrease of its frequency, but none ever regained normal digestive functional states.

Pain was experienced as a continuous or intermittent symptom and could usually only be relieved with high doses of analgesics.

It feels as if you had a mucositis, and the skin is gone … and then food rides over it. (P3, Pos. 79)

Some participants did not suffer from pain at all.

Moreover, the participants often suffered from a lack of appetite or nausea, followed by a significant weight loss, which caused great distress. Some participants felt an inner barrier, making them unable to eat.

You can’t eat. Or you eat a little bit and that was it. It is not possible. That’s all, you cannot describe it. (P12, Pos. 95)

#### Severe GvHD of the skin

Severe skin GvHD caused restlessness due to pain and pruritus. It was associated with visual stigmatization, hence insecurity in public, which caused a feeling of shame and disgust toward oneself.It was perceived as onerous that therapies could not bring immediate relief due to the delayed effects of topical therapies. For the application, the participant felt dependant on HCPs.

#### Severe GvHD of the lung

One participant suffered from pulmonary GvHD, which manifested as a pronounced weakness due to chronic breathlessness. The participant reported about the permanent limitation of his everyday life, needing daily assistance by family members and a wheelchair when leaving the house. The GvHD compromised recovery from the allo-HSCT procedure.

#### Further forms of GvHD

Contributing to the overall burden of the participants, various – although often scored as milder – organ affections of GvHD (liver/bile, joints/fascia, eyes, and oral/genital mucosa) were endured by the participants. These were predominantly perceived as bothersome and another side to be taken care of, but less dangerous than the severe forms of GI, skin, and pulmonary GvHDs.

### Overall burden associated with severe GvHD

#### Weakness

The constant presence of weakness limited the participants’ activities of daily living (ADLs) and made participants feel helpless.

I couldn’t really do anything that would build up my body because I was always short of air. (P11, Pos. 32)

Particularly muscle weakness was limiting as patients had difficulties judging whether strength was sufficient for certain ADLs. They described a tendency to fall, inability to get up after falling, incapability to stand, walk, or climb stairs, or a limitation of these functions.

#### Isolation

During in-hospital stays, isolation and loneliness were mentioned as a decisive burden, particularly on the BMT ward, where patients are more isolated for protective measures, as compared to regular wards. Since most of the participants were hospitalized for a long time, they often only saw 1 or 2 authorized visitors and missed their social network. Participants imagined this to be particularly burdensome for patients without family support.

I have seen those people. Young people that have died. Because they have just been so lonely. They have lived alone. (P12, Pos. 101)

The persisting high symptomatic load after hospital discharge, e.g., weakness or fecal incontinence, isolated the participants from their social environment and limited their participation in public life. Changes of social relations were noted and the loss of former close relationships saddened many participants.

#### Dependence on others

Feeling dependant on others and needing assistance with ADLs was uncomfortable for the participants and made them feel embarrassed and vulnerable, particularly if being cared for by HCPs and not family members.

So, when you lay in bed and realize, okay, I have to go to the toilet, but you cannot manage it at all. And then you have to be washed, by people who are actually strangers to you. This has been the worst for me. (P11, Pos. 10)

A feeling of losing control emerged as well as the concern of being a burden, while not being able to fulfill the (expected) social function.

#### Mental strain due to GvHD

The participants had not expected to get such severe GvHD and had difficulties to mentally cope with it. It was straining not to be able to participate properly in life for such a long time and sometimes the extent to which the allo-HSCT can be considered curative was questioned.

It was a disaster, or still is. I still can’t cope with it at all. Um, apart from the fact that the question always is what does it mean to be cured of the underlying disease? Is that really the case? I have no idea. (P10, Pos. 35)

#### Depressive symptoms and existential distress

Symptoms of depression often occurred when participants were in very poor physical conditions, felt overwhelmed by new complications, or realized health limitations could become chronic. Particularly a lack of prospect of recovery caused hopelessness and made the participants feel desperate, powerless, and demoralized. Further triggers were pain, exhaustion, loneliness, feeling locked away, reduced ADL, and reduced working abilities. Some participants were diagnosed with depression and several were treated with antidepressants.

Most participants experienced tiredness of living or a desire to die when the above-mentioned triggers peaked. These thoughts could be associated with fear, but had a liberating character in burdensome phases.

This comes up sometimes and then I tell myself: Before living like this, I prefer not to live at all. (P13, Pos. 66)

In 2 cases, participants reported they had suffered from active suicidal ideation because of their GvHD burden.

#### Uncertainty and fear

The occurrence of the GvHD made the participants aware of the unpredictability of their prognosis. This generated uncertainty and fear, such as the fear of persistence or recurrence of GvHD symptoms or the recurrence of the underlying disease.

I think I was even more afraid of that than of dying. That I would somehow have to be … yes … cared for all my life or something. (P7, Pos. 55)

At a certain point, the treatment options were also perceived as unclear and “only trial and error” (P10, Pos. 57), which next to uncertainty caused fear of limited treatment options.

Other important fears included the uncertainty of managing life independently, becoming permanently dependant on others, getting complications which cause rehospitalization, or the permanent need of medication with harmful side effects.

#### Perception of the threat to life

Apart from life-weary thoughts, which we classified as an expression of psychological distress, all participants reported to perceive the life-threatening character of their diagnosis, mainly during inpatient-stays, but in form of a dynamic process with individual peaks. Being confronted with the death of other transplant recipients increased anxiety and the feeling of being in a life-threatening situation.

A minority of patients described having felt very close to dying at some point and described acceptance and a sense of letting go. But participants mostly did not feel close to dying.

Well, I knew that all of this can be life-threatening, but I thought no, it won’t be. (P5, Pos. 60)

Most participants described thoughts of the threat to life as phase-dependent, but one participant saw death as a constant threat, causing consistent fear and despair.

#### Cognitive impairment

A reduced ability to focus and to remember or difficulties in carrying out familiar work processes were described, which made participants feel easily overloaded. However, these impairments usually improved over time.

### Resources/coping factors

Being cared for by family members and friends was seen as the most important resource to cope with burdens caused by GvHD.

A feeling of hope was given by doctors who expressed an optimistic attitude toward a positive recovery outcome.

Yes, but Dr Y told me, everything will be fine, Miss X. and, when I heard that, I thought in this moment okay, then I will not give up. (P5, Pos. 2)

The participants perceived the offer of psychotherapy as relieving and it was seen as a good opportunity to speak freely, regardless of its use.

If being religious (all religious participants were of Christian faith), pastoral care seemed to serve the same function as psychotherapy. Faith was seen as a resource, but doubts were frequently experienced alongside hope, particularly in tough times.

A positive mind set was addressed as a very important coping factor, while establishing it was considered as a lengthy process that may not be possible during worst times.

She told me, you have to make this disease your friend. … I did not understand this at all … but in the meantime … in the meantime I think I understand what she meant. That is the only possibility to somehow get into a light-hearted life. By accepting and living with the disease. Whether I make it my friend … (person laughs out loud), this is still difficult. But I understand where one has to get to. (P1, Pos. 78)

Many participants described that the resource of setting future goals was important, but only possible in an improved state of health. During symptom peaks, participants stated that they often did not think about the future and lived from day to day.

### Support needs

The participants had a great need for medical information. Due to the unpredictability of the disease course, they felt very insecure and longed to be able to assess their health status and to gain control over their situation.

Structural needs often related to ADLs after discharge, e.g., in form of domestic help. These were particularly expressed by patients without family support.

### The course of disease

The disease courses of the participants were heterogenous and unpredictable, but all characterized by long inpatient stays and frequent hospital readmissions due to complications.

Directly in the BMT Ward, um. One or two months, I think. But then I had several complications and … I believe altogether it was about a year that I was here. (P8, Pos. 18)

After discharge, none of the participants could live without assistance, which caused despair and the feeling of being a burden. Activity levels were extremely reduced and it took months or even years to return to an autonomous life.

At the beginning, I couldn’t do much, I have to say. …. During the day I just laid here on the sofa. …. And then last year we walked up and down the street a bit. That’s all I managed to do. (P2, Pos.13)

#### Resuming work

Return to work took place at least 2 years after transplantation, when the participants felt more stable. The majority of participants considered themselves not (fully) able to work.

#### Quality of life

For none of the participants, the QoL reached the level before the diagnosis of the underlying disease and most suspected this would not happen anymore, which was unexpected and difficult to accept. QoL was worst during hospitalization and while experiencing high symptomatic burden.

At zero. I don’t have any quality of life. You don’t have that with diarrhea. And when you can’t participate in life, not at all. (P3, Pos. 84)

### Perception of PC

Most participants did not know what the term PC meant. In case they knew about it, PC was mostly perceived as end-of-life care. In this case, the question of whether they had received PC evoked irritation or defensiveness.

Only some of the participants were aware of having received specialist PC, which was positively associated with pain-relieving therapy. Most participants were not sure about having received specialist PC or could not distinguish between specialist PC and other specialties.

## Discussion

Our qualitative study emphasizes burden, resources, and needs of patients with severe GvHD. Next to specific symptoms of the severely affected organs, the participants suffered from long and mostly incomplete recovery processes, riddled with occurrences of somatic distress, complications, and frequent hospitalizations.

We want to highlight the unpredictability of the GvHD course in our cohort, which was perceived as particularly burdensome among the patients, causing a constant threat and a feeling of demoralization. They had not expected such a severe complication and felt shocked how much it suddenly dominated their lives. This corroborates with another qualitative study, which explored QoL after GvHD in the UK and named the unpredictability as burdensome, leading to uncertainty about the future (de Vere Hunt et al. 2021).

Participants named prolonged isolation as an important source of suffering. This came from protective isolation within prolonged hospital stays, which is known to cause loneliness and psychological distress, and poor post-discharge health, and is consistent with our findings (Biagioli et al. 2017; Tecchio et al. 2013). The participants in our study felt very dependent on their HCPs, but also on family members. This was linked to extreme physical weakness, which impaired their ability for self-care. While reduced physical functioning is a known restriction for patients with GvHD (Agh et al. 2019; Baird et al. 2013; Pidala et al. 2013), we would like to emphasize its high extent among our cohort, resulting in all of them requiring comprehensive assistance after discharge. Having been interviewed at different stages of survivorship, most participants described a protracted, undulating, but progressive recovery process. All of them described a persistently reduced QoL and did not anticipate that the pretransplant level could ever be reached again, what we consider to be a central message of this study. This is supported by several studies, which found severe cGvHD to be associated with very poor QoL (Baird et al. 2013; Kurosawa et al. 2017; Mo et al. 2013; Pidala et al. 2011).

Regarding returns to their occupational work, a long sick leave of at least 2 years was remarkable among the participants if a return to work was possible at all and most of the participants then worked part time. An association between suffering from aGvHD or cGvHD and taking full-time sick leave for 1 year after transplantation has been already described (Bhatt et al. 2021; Eriksson et al. 2021). An investigation of the correlation between the grade of severity of GvHD and the average time of sick leave would be informative.

Almost all of the participants reported symptoms of depression and the majority felt tired of living during the peaks of symptomatic burden. Two studies found that approximately 1/5–1/3 of patients with cGvHD suffer from depressive symptoms and that its appearance may be linked to a higher burden of cGvHD (El-Jawahri et al. 2018b; Jacobs et al. 2019). We hypothesize that severe GvHD could be considered a trigger for psychological distress, as it prolongs the course of allo-HSCT, is often unexpected in its dimension, and must be managed in an already health-reduced state. Research on the desire to die in the context of GvHD does not seem to exist yet. But research on the broad term of “desire to die” has intensified in recent years for people suffering from life-limiting progressive diseases. A first definition published in the German Palliative Care Guideline describes “desire to die” as a continuum ranging from acceptance of death (satiety of life) to acute (consciously planned) suicidality. Between these 2 poles, the pressure to enact the desire increases, hoping to die soon or wishing to accelerate the dying phase (Kremeike et al. 2021). The statements of the participants in this study are reflected in the above definition. Further research in this field is indicated.

Among the participants, it was common to associate the term PC with end-of-life care. They were not sufficiently informed about this specialty, which aims to improve QoL not only near the end of life, but also more generally in serious health-related suffering due to severe illness (Radbruch et al. 2020). Furthermore, not all participants were given the opportunity to receive a PC treatment. This fits with the current state of research, according to which the use of PC in allo-HSCT is scarcely investigated and practiced (Pralong et al. 2023; Simon et al. 2021).

As for many patient groups, social resources were very important to the participants (Applebaum et al. 2014; Nørskov et al. 2021). Alarmingly, they considered the services provided by the German health care system as not sufficient for patients without a supportive social system, which also had been pointed at in another qualitative study from Germany, examining the needs after allo-HSCT (Parisek et al. 2021).

Finally, a need for medical information was frequently addressed due to the unpredictability of the course of disease and the wish to gain control over the situation.

## Limitations

The majority of the participants suffered from severe GvHD of the GI, which means that the sample is not representative of all manifestations of severe GvHD. In addition, there may be a participation bias as some patients were too ill to participate in the study and a recall bias if interviewed several years after allo-HSCT. Furthermore, some topics such as sexual dysfunction might not have been addressed by the participants. It might be associated with shame. In 2 interviews, family members were present, which could have restricted the unbiased verbalizations by those 2 participants. It should be noted that this study lacks racial diversity as all participants were Caucasian in Germany. Furthermore, it was conducted in a European care setting and may not be transferable to other settings.

## Conclusion

This qualitative study provided an overview of the complexity and burden that participants have to endure due to severe GvHD, further complications, and the unpredictability of their course. The frequently addressed desire to die in the context of severe GvHD has not yet been described in this detail and represents an important field of future research.

Participants’ definition of the term of PC as an end-of-life care suggests that the meaning of PC in the German society remains misinterpreted or is unknown. This study appears to be the first to make statements on PC in the context of GvHD in the German healthcare system, which shows a lack of research on its local use. Since our findings support that GvHD symptoms represent an important need for timely PC involvement, further research in this field is highly warranted.
